# Supplementary material for: Host-driven temperature dependence of Deformed wing virus infection in honey bee pupae
Source: Commun Biol. 2023 Mar 27;6:333. doi: 10.1038/s42003-023-04704-6 (PMC10042853; doi:10.1038/s42003-023-04704-6)
Supplement: Supplementary file 2 — Supplementary Information [file 42003_2023_4704_MOESM2_ESM.pdf]

Host-driven temperature dependence of Deformed wing virus infection in honey bee pupae (Supporting information)

Evan C Palmer-Young [1]*, Eugene V Ryabov [1,2], Lindsey Markowitz[1,3], Dawn L Boncristiani [1], Kyle Grubbs [1], Asha Pawar [1], Raymond Peterson [1], Jay D Evans [1]

[1] USDA-ARS Bee Research Laboratory, Beltsville, MD, USA

[2] Department of Entomology, University of Maryland, College Park, MD, USA

[3] Department of Biology, University of Maryland, College Park, MD, USA

*Corresponding author: ecp52@cornell.edu, evan.palmer-young@usda.gov

SUPPORTING INFORMATION

Supplementary Note 1. Amino acid sequences for DWV protease and fluorogenic substrate and composition of protease reaction buffer.

Sequence for DWV 3C Protease

GSTQQVDAAVNKILQNMVYIGVVFPKVPGSKWRDINFRCLMLHNRQCLMLRHYIESTAAFPEGTKYYFKYIHNQETR
MSGDISGIEIDLLNLPRLYYGGLAGEESFDSNIVLVTMPNRIPECKSIIKFIASHNEHIRAQNDGVLVTGDHTQLLAFENNN
KTPISINADGLYEVILQGVYTYPYHGDGVCGSILLSRNLQRPIIGIHVAGTEGLHGFGVAEPLVHEMFTGKA

Sequence for FRET Peptide

(DABCYL)-VQAKPEMDNPNPG-(Glu(EDANS))

Protease Reaction Buffer

Tris, pH 7.0	50 mM
NaCl	150 mM
EDTA	1 mM
Dithiothreitol	1 mM
Glycerol	10%
