# Supplementary material for: Host-driven temperature dependence of Deformed wing virus infection in honey bee pupae
Source: Commun Biol. 2023 Mar 27;6:333. doi: 10.1038/s42003-023-04704-6 (PMC10042853; doi:10.1038/s42003-023-04704-6)
Supplement: Supplementary file 3 — Description of Additional Supplementary Files [file 42003_2023_4704_MOESM3_ESM.pdf]

Description of Additional Supplementary Files

File Name: Supplementary Data 1

Description: Zipped folder with data spreadsheets for protease activity, bee traits, and pupal infection.
